# Supplementary material for: Enumerating the economic cost of antimicrobial resistance per antibiotic consumed to inform the evaluation of interventions affecting their use
Source: Antimicrob Resist Infect Control. 2018 Aug 9;7:98. doi: 10.1186/s13756-018-0384-3 (PMC6085682; doi:10.1186/s13756-018-0384-3)
Supplement: Supplementary file 1 — Table S1. Standard units per course by antibiotic drug class. Table S2. Summary of assumptions and limitation. (DOCX 22 kb) [file 13756_2018_384_MOESM1_ESM.docx]

**Additional file 1**

**Table S1**. Standard units per course by antibiotic drug class

| **Indication** | **Antibiotic**  **Drug class** | **Prescribed Dosage** | **Available form** | **Number of pill/vial/tablets per day** | **Duration (Days)** | **SUs per course** |
| --- | --- | --- | --- | --- | --- | --- |
| Community acquired pneumoniae | Levofloxacin (Quinolone) | 500mg twice daily | 250mg tablets | 4 | 7 | 28 |
| Syphilis | Ceftriaxone (Cephalosporin) | 1g daily | 1g vials | 1 | 14 | 14 |
| Severe dental infections | Co-amoxiclav (Broad spectrum penicillin) | 250mg thrice daily | 250mg tablets | 3 | 5 | 15 |
| Serious Gram-negative infections resistant to gentamicin | Amikacin (Aminoglycoside) | 15 mg/kg daily | 250mg/mL, 2mL vials | 2 | 10 | 20 |
| Clostridium difficile infection | Vancomycin (Glycopeptide) | 125mg four times daily | 125mg capsules | 4 | 14 | 56 |
| Streptococcal infection | Phenoxymethylpenicillin (Narrow spectrum penicillin) | 250mg four times daily | 250mg tablets | 4 | 10 | 40 |
| Nosocomial Pneumonia | Meropenem (Carbapenem) | 1g IV thrice daily | 1g vials | 3 | 7 | 21 |
| Community-acquired pneumonia, low to moderate severity | Azithromycin (Macrolide) | 500 mg once daily for 3 days | 500mg tablets | 1 | 3 | 3 |

**Table S2**. Summary of assumptions and limitation

| **Assumption / Limitation** | **Discussion** | **Likely Impact** |
| --- | --- | --- |
| **Pertaining to Cost data** | | |
| The model only takes into account the impact of mortality and direct medical costs attributable to resistant infections. | It excludes other possible direct and indirect effects such as the spread of resistant infections, other medical services that will be affected by loss of effective prophylactic treatment | Underestimating the economic cost of AMR |
| Only present burden is accounted for. | AMR is predicted to continue to spread, incurring ever higher tolls in future years. Models to predict and quantify the future burdens are impractical. | Underestimating the economic cost of AMR |
| The model takes into account only a subset of resistant infections. | Further data on the incidence of other resistant infections and their associated costs could be incorporated if/when available | Underestimating the economic cost of AMR |
| One death was assumed to result in 10 life years. | Data on mean number of productive life years lost due to an AMR associated death are limited. | Under- or over-estimation of the economic loss data per organism. |
| The drug classes implicated could be responsible for selective pressure in other excluded organisms. | Antibiotic consumption is likely to drive resistance in a host of other pathogens for which mortality estimates are less readily available | Underestimates the economic cost of AMR attributable to these drugs. |
| Assumption that selected drug classes contribute equally in promoting the resistance. | There are complex mechanisms involving propagation of resistance. Hence, relative contribution intuitively, should be different. | The model can use weighing to incorporate relative contributions once better evidence is available. |
| The consumption data is for 2014, as this was the latest data available. | More recent consumption data for the countries would have been ideal. | If consumption increased from our figures, economic cost might have been overestimated, and vice versa. |
| The consumption data was estimated using retail and/or hospital information.^[[1]](#footnote-1)^ | The actual consumption at the patient-level could be much lower than this estimate. | Could contribute to an underestimation of the economic cost. |
| The data used for resistance only represents blood samples.^[[2]](#footnote-2)^ | Inclusion of other samples would make the data richer if available for all countries. | Including other samples could increase or decrease the rates and henceforth shift the cost in either direction. |

1. Van Boeckel TP, Gandra S, Ashok A, Caudron Q, Grenfell BT, Levin SA, et al. Global antibiotic consumption 2000 to 2010: An analysis of national pharmaceutical sales data [supplementary appendix] [Internet]. Vol. 14, The Lancet Infectious Diseases. 2014 [cited 2016 Aug 10]. p. 742–50. Available from: http://www.thelancet.com/cms/attachment/2021722899/2041540096/mmc1.pdf [↑](#footnote-ref-1)
2. The Center for Disease Dynamics Economics and Policy. ResistanceMap beta [Internet]. [cited 2016 Jun 22]. Available from: http://resistancemap.cddep.org [↑](#footnote-ref-2)
